# Neocortex neurogenesis and maturation in the African greater cane rat

**DOI:** 10.1186/s13064-023-00175-x

**Published:** 2023-10-13

**Authors:** Oluwaseun Mustapha, Thomas Grochow, James Olopade, Simone A. Fietz

**Affiliations:** 1 Neuroscience Unit, Department of Veterinary Anatomy, College of Veterinary Medicine, Federal University of Agriculture Abeokuta, Abeokuta, Ogun State Nigeria; 2 Institute of Veterinary Anatomy, Histology and Embryology, Faculty of Veterinary Medicine, University of Leipzig, Leipzig, Germany; 3 Neuroscience Unit, Department of Veterinary Anatomy, Faculty of Veterinary Medicine, University of Ibadan, Ibadan, Oyo State Nigeria

**Keywords:** Greater cane rat, Grasscutter, Neocortex development, Neurogenesis, Neuron maturation, Precocial, Altricial

## Abstract

**Background:**

Neocortex development has been extensively studied in altricial rodents such as mouse and rat. Identification of alternative animal models along the “altricial-precocial” spectrum in order to better model and understand neocortex development is warranted. The Greater cane rat (GCR, *Thyronomys swinderianus*) is an indigenous precocial African rodent. Although basic aspects of brain development in the GCR have been documented, detailed information on neocortex development including the occurrence and abundance of the distinct types of neural progenitor cells (NPCs) in the GCR are lacking.

**Methods:**

GCR embryos and fetuses were obtained from timed pregnant dams between gestation days 50–140 and their neocortex was analyzed by immunofluorescence staining using characteristic marker proteins for NPCs, neurons and glia cells. Data were compared with existing data on closely related precocial and altricial species, i.e. guinea pig and dwarf rabbit.

**Results:**

The primary sequence of neuro- and gliogenesis, and neuronal maturation is preserved in the prenatal GCR neocortex. We show that the GCR exhibits a relatively long period of cortical neurogenesis of 70 days. The subventricular zone becomes the major NPC pool during mid-end stages of neurogenesis with Pax6 + NPCs constituting the major basal progenitor subtype in the GCR neocortex. Whereas dendrite formation in the GCR cortical plate appears to initiate immediately after the onset of neurogenesis, major aspects of axon formation and maturation, and astrogenesis do not begin until mid-neurogenesis. Similar to the guinea pig, the GCR neocortex exhibits a high maturation status, containing neurons with well-developed dendrites and myelinated axons and astrocytes at birth, thus providing further evidence for the notion that a great proportion of neocortex growth and maturation in precocial mammals occurs before birth.

**Conclusions:**

Together, this work has deepened our understanding of neocortex development of the GCR, of the timing and the cellular differences that regulate brain growth and development within the altricial–precocial spectrum and its suitability as a research model for neurodevelopmental studies. The timelines of brain development provided by this study may serve as empirical reference data and foundation in future studies in order to model and better understand neurodevelopment and associated alterations.

**Supplementary Information:**

The online version contains supplementary material available at 10.1186/s13064-023-00175-x.

## Background

The neocortex is a unique and highly divergent portion of the mammalian brain. It plays a key role in commanding higher functions such as sensory perception, emotion, voluntary motor control and cognition. It is composed of two major classes of neurons: glutamatergic projection neurons (approximately 80%) and GABAergic interneurons (approximately 20%), which are organized into six layers. Projection neurons are mainly born in the dorsal telencephalon and migrate radially to reach their destination in the cortical plate (CP) in a birth date-dependent *inside*-*out manner*, whereas the majority of interneurons are born in the ventral telencephalon and migrate tangentially into the developing neocortex [42, 47, 69, 76, 130].

The overwhelming majority of neocortical neurons are generated during prenatal development. Projection neurons are derived from two distinct types of neural stem and progenitor cells (NPCs): apical progenitors (APs) and basal progenitors (BPs) [5, 24, 32, 84, 126, 127]. The cell body of APs resides in the ventricular zone (VZ), the germinal zone adjacent to the lateral ventricle. APs exhibit a radially oriented basal process, characteristically express the transcription factor Pax6 and expand by symmetric proliferative divisions prior to neurogenesis [39, 61, 81, 101, 102]. With the onset of neurogenesis, the majority of them divides asymmetrically, thereby giving rise to BPs that populate the subventricular zone (SVZ), the germinal zone basal to the VZ [45, 82, 91]. BPs consists of two major subtypes: basal intermediate progenitors and basal radial glia [31, 44, 104]. Basal intermediate progenitors retract their process before mitosis and characteristically express the transcription factor Tbr2. They represent the major BP cell type in mouse and rat, in which they mainly divide symmetrically into two neurons and therefore display limited proliferative potential [7, 30]. Basal radial glia share key features with APs including the radially oriented process and expression of Pax6 and are able to undergo repeated cell division. They represent the major BP cell type in primates that exhibit a high degree of neocortex expansion [12, 31, 44, 60, 104]. Contrary to mouse and rat, a major proportion of basal intermediate progenitors in primates is characterized by sustained expression of Pax6 and a high proliferate potential. This high abundance of proliferative BPs results in an expanded SVZ and a high neuronal output in primates, especially in human [12, 18, 31, 33, 35, 44, 95].

Following their migration into the CP, neurons undergo maturation and differentiation in order to ensure proper neural circuit assemblage and connectivity. This meticulously-controlled event is characterized by the acquisition of morphological and electrophysiological capacities such as dendrite morphogenesis and axonal sprouting [36, 41, 50, 83, 129] and requires the presence of distinct support and supply structures, i.e. glial cells for providing nutrients and oxygen, the myelination of axons, the development of the blood–brain-barrier and the homeostasis of the extracellular milieu [41, 83, 129].

Neocortex development has been extensively studied in laboratory rodents, most of them being born in an altricial state [65, 76, 108]. The offspring of highly altricial mammals are born in an immature state as they are usually relatively immobile, lack hair, possess closed eyes, and are unable to obtain food on their own at birth. Whereas the offspring of precocial species are relatively mature and mobile, possess a well-developed coat, open eyes and require little food from the mother at birth [13, 25, 26, 77, 127]. There is increasing evidence suggesting that precocial and altricial species show clear differences in the pattern of prenatal neurogenesis and brain maturation [4, 15, 19, 27, 40, 59, 71, 79, 97, 121, 124]. In order to better understand neocortex development and evolution, there is a growing awareness of the need to examine alternative animal models along the “altricial-precocial” spectrum [6, 56, 62, 96].

The greater cane rat (GCR, *Thyronomys swinderianus*) also referred to as the grasscutter is an indigenous precocial African rodent [2, 111]. It belongs to the suborder Hystricomorpha and inhabits Sub-Saharan reed-beds and riverbanks. The GCR is the fourth largest extant rodent worldwide with an average body weight of 4 kg and a long gestation period of 150 days [1, 28, 56, 57, 89, 111]. Although basic aspects of the prenatal and early postnatal brain development in the GCR have been documented [16, 52, 53], detailed information on neocortex development including the presence and abundance of the distinct types of NPCs in the GCR are lacking. Therefore, this study aims at elucidating the prenatal development of the neocortex in the GCR and at correlating these findings with existing data in the literature of closely related precocial and altricial species, i.e. guinea pig and dwarf rabbit.

Here, we show that the main period cortical neurogenesis in the GCR takes place during mid-end gestation, followed by prenatally initiating processes of gliogenesis and neuron maturation resulting in a highly mature neocortex at birth. We provide initial baseline timelines on the prenatal neocortex development and maturation in the precocial GCR, which could serve as important empirical reference data in future studies, especially in neural stem cell culture experiments and ecotoxicological studies. Together, our findings provide important insights into the timing and the cellular differences that regulate brain growth and development within the altricial–precocial spectrum.

## Methods

### Animals and brain samples

Embryos and fetuses were obtained from timed pregnant GCR does used in a previous study [86]. In brief, does were obtained from commercial farms (Onileola Farms®, Osun State and Pavemgo Farms®, Southwest, Nigeria) and housed in 80 cm × 40 cm × 35 cm concrete cages. Water and elephant grass (*Pennisetum purpureum*) were given ad libitum and supplemented with cassava and fresh maize. Four nulliparous females were housed together with one male. Successful natural mating was indicated by post-pairing perineal changes [86]. Females were carefully examined twice a day. On the observation of these signs, females were immediately separated from the male and the date of appearance of mating is recorded as the first day of gestation. Successful pregnancy was determined by abdominal palpation and further validated sonographically using a portable ultrasound machine (Kaixin KX 2000^R^, GmbH, Ellfestrass, Hamburg, Germany), with a 5.0 MHz transducer fitted with a 3.5 MHz curvilinear and 7.0 MHz linear transducers. All experiments were performed in accordance with the University of Ibadan Animal Care and Use Research Ethics Committee (UI-ACUREC),reference number: UI-ACUREC/17/0066.

Pregnant does were anaesthetized with an intraperitoneal injection of ketamine hydrochloride (100 mg/kg; Ketanir®, Gujarat, India) and xylazine hydrochloride (10 mg/kg; Xylazine 20 Inj® Kepro, Holland) between gestation days (GD) 50–140. Embryos and fetuses (GD50, *n* = 2; GD60, *n* = 2; GD70, *n* = 4; GD80, *n* = 3; GD100, *n* = 6; GD110, *n* = 4; GD120, *n* = 2; GD130, *n* = 2; GD140, *n* = 4) were explanted via caesarean section [86]. Whole embryos at GD50 were fixed immediately in 4% paraformaldehyde (PFA), washed in phosphate-buffered saline (PBS) and stored in 70% ethanol at 4 °C until processing. Brains of GD60-GD140 embryos/fetuses were carefully dissected, fixed in 4% PFA for 2 days at 4 °C, briefly washed in PBS and stored in 0.02% PBS azide at 4 °C. Before further processing, images of the dorsal view were acquired using a digital camera (Sony DSCW830 20.1 MP, Japan).

### Immunocytochemistry

Whole fixed embryos of GD50 and cerebral hemispheres of GD60-140 GCR were used for cryosectioning. Samples were cryoprotected in 30% sucrose in PBS on a rocker (ROTH, JKIKA Labortechnik, HS250 Basic, Germany) at room temperature until they sank to the bottom. Sucrose solution was then replaced with Tissue-Tek (Sakura Finetek, Staufen im Breisgau, Germany) for 2 h and samples were transferred into a peel-a-way disposable plastic tissue embedding molds (R-40) (22 mm × 40 mm × 20 mm; Polysciences Inc., USA) containing Tissue-Tek. Molds were placed in a tyrofoam containing dry ice, allowed to solidify, and preserved at − 20 °C until use. Cryosections were sliced at 20 μm for GD50 and 30 μm for GD60-140. Serial coronal sections of the complete telencephalon were cut. Mid-ventricular sections along the rostro-caudal axis were used for the immunohistochemical staining.

Immunohistochemistry was carried out as described previously [31]. Primary antibodies were incubated overnight at 4 °C and secondary antibodies were incubated for 1 h at room temperature. The following primary antibodies were used: Pax6 (1:100, rabbit, Biozol, Eching, Germany, BLD-901301), Tbr2 (1:100, sheep, R&D Systems, Abingdon, United Kingdom, AF6166), Tbr1 (1:200, rabbit, Millipore, Darmstadt, Germany, AB10554), MAP2 (1:100, chicken, Abcam, Amsterdam, Netherlands, ab5392), Hu C/D (1:100, rabbit, Abcam, Amsterdam, Netherlands, ab96474), neurofilament (NF) (1:100, rabbit, Abcam, Amsterdam, Netherlands, ab204893), GFAP (1:225, rabbit, Antibodies, Cambridge, United Kingdom, A85419), Olig2 (1:100, mouse, Abcam, Amsterdam, Netherlands, ab236540), MBP (1:100, rat, Abcam, Amsterdam, Netherlands, ab7349). Secondary antibodies coupled to Alexa Fluor 488, 555, 647 (1:500, Life Technologies, Darmstadt, Germany) were used. All sections were counterstained with DAPI (1:500, Sigma Taufkirchen, Germany), mounted with Mowiol (Merck Biosciences, Darmstadt, Germany), coverslipped and kept at 4 °C.

### Image acquisition, cell counting and statistical analysis

Immunofluorescence images were captured with a Leica SP8 confocal laser-scanning microscope (× 20/0.75 W or × 40/1.1 W objectives) (Leica Microsystems, Mannheim, Germany). All images were obtained as single-optical sections. The VZ, SVZ, intermediate zone (IZ)/subplate (SP) and cortical plate (CP) were identified based on their cytoarchitecture as described previously [105]. In brief, the VZ was defined as the layer of tightly stacked cell with radial nuclei lining the lateral ventricle. The SVZ was identified as the layer adjacent to the VZ with less tightly stacked and relatively sparser cells compared to VZ. The IZ/SP was delineated as the cell layer between the SVZ and CP with very low cell density. The CP was identified as a layer of densely packed cells dorsal to the IZ/SP.

Images were processed with Fiji software. Quantitative analysis of individual cell counts was done with Fiji software using a Multiclass Cell Counter plugin [106]. Positive nuclei for the parameters indicated were counted in rectangular sectors of the cortex spanning its entire thickness (Hu C/D, Olig2) or the thickness of the VZ or SVZ/IZ (Pax6, Tbr2) and expressed as number of cells per 100 µm ventricular surface. The fluorescence signal of single channels was counted using grayscale color and an adjusted threshold. All quantifications were performed on images from the dorsolateral telencephalon. Radial thickness of the germinal zones and length of the ventricular surface were determined by tracing it in Fiji software. Data obtained were displayed using Prism software 7.0 (GraphPad Software Inc., San Diego, USA).

Linear regression analysis and correlation analysis using Pearson correlation coefficient were performed using Prism software 6.0 (GraphPad Software Inc., San Diego, USA). Data for neurogenic period and gestation length of guinea pig and dwarf rabbit were obtained from the literature [59]. Data for neurogenic period of GCR were analyzed in this study.

## Results

### Onset and duration of neurogenesis in the prenatal GCR neocortex

**Fig. 1:**
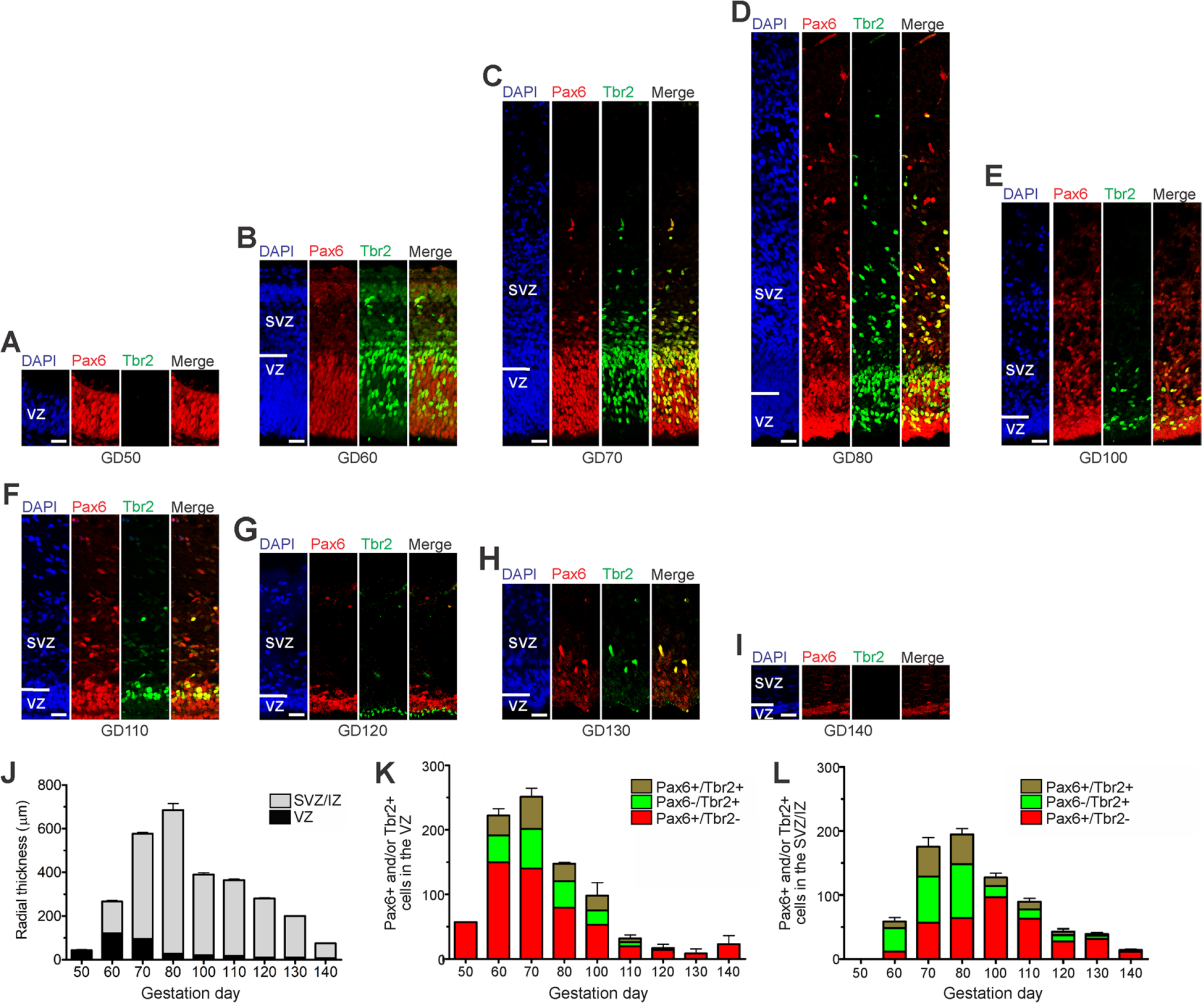
Pax6 and Tbr2 expression in the germinal zones of the developing GCR neocortex. **A–I** Double-Immunofluorescence for Pax6 (red) and Tbr2 (green) and DAPI staining (blue) on 20 µm cryosections of GD50 and 30 µm cryosections of GD60-140 GCR neocortex. Scale bars, 25 µm. **A, B** The entire cortical is shown. **C–I** The top margin of the image corresponds to the transition zone SVZ/intermediate zone. **J** Quantification of the radial thickness of the germinal zones in the developing GCR neocortex. **K, L** Quantification of Pax6 + /Tbr2– (red), Pax6 + /Tbr2 + (brown) and Pax6–/Tbr2 + (green) NPCs in the VZ (**K**) and SVZ (**L**), expressed as number of cells per 100 µm ventricular surface. Cortical wall corresponding to a total ventricular surface of 309–1426 µm was analyzed. (**J–L**) Data represent mean ± SEM and are from two fetuses each. VZ, ventricular zone; SVZ/IZ, subventricular zone/intermediate zone; CP, cortical plate

We first examined the development of the germinal zones and the NPCs they contain in the prenatal GCR neocortex by immunohistochemistry using markers for the transcription factors Pax6 and Tbr2, that are characteristically expressed by distinct mammalian NPCs [30, 31, 44, 103]. At GD50, the GCR neocortex consists of a relatively thin VZ made up of NPCs that are solely Pax6 + (Fig. 1A). Tbr2 + NPCs in the GCR VZ appeared first at GD60 (Fig. 1B) and persisted until GD130 (Fig. 1C-H). At all stages analyzed, Tbr2 + NPCs constitute less than 50% of all NPCs in the GCR VZ (Fig. 1K). The number of Pax6 + /Tbr2– APs and the VZ thickness reach a maximum at GD60 and progressively decline thereafter (Fig. 1J, K). By GD130, the VZ is reduced to a single-cell layer, the putative ependymal layer (Fig. 1H).

**Fig. 2:**
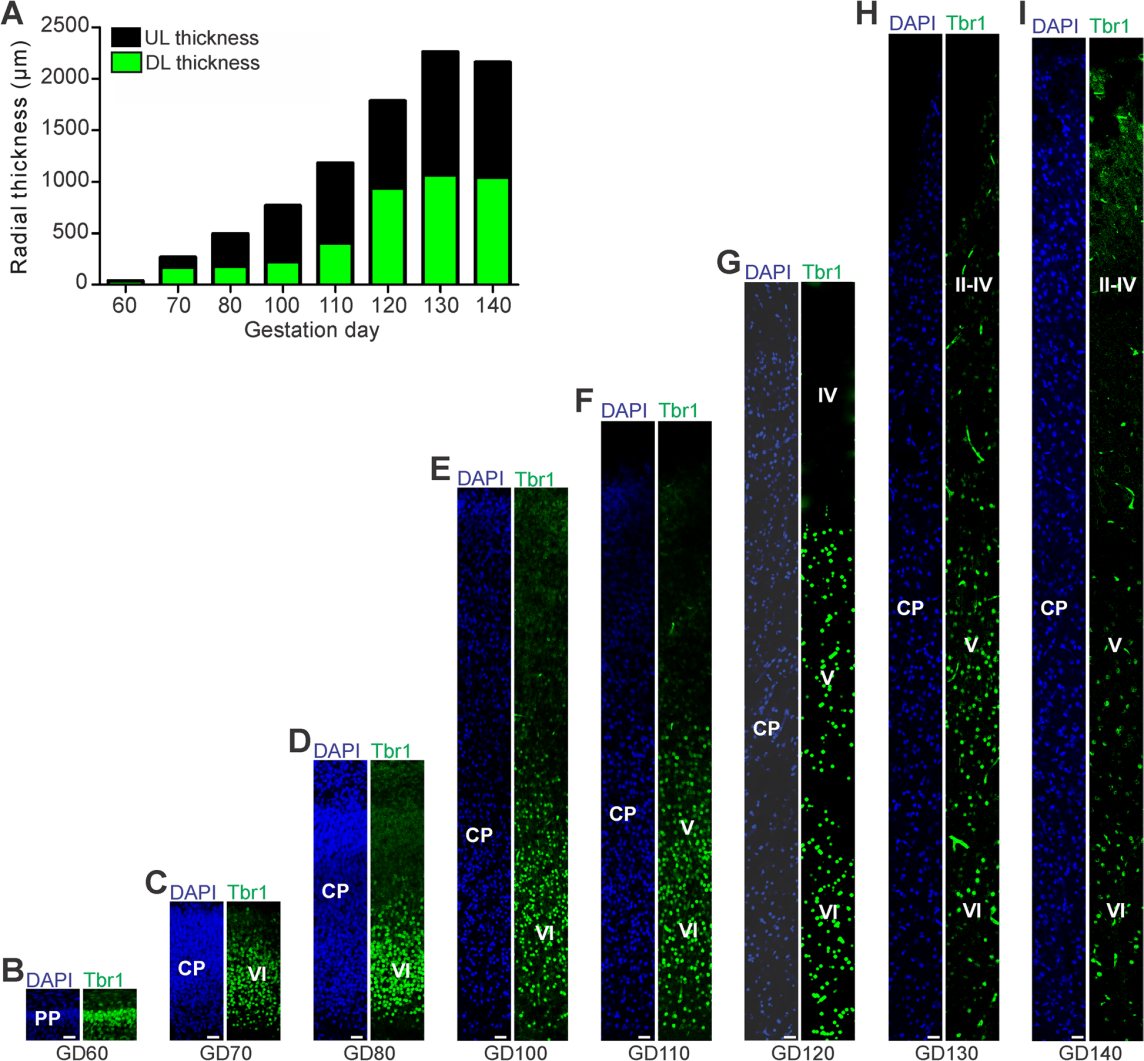
Tbr1 expression in the cortical plate of the developing GCR neocortex. **A** Quantification of radial thickness of the upper (Tbr1–) and deeper (Tbr2 +) layers of the developing neocortex. Data are from one fetus each. **B–I** Immunofluorescence for Tbr1 (green) and DAPI staining (blue) on 30 µm cryosections of GD60-140 GCR neocortex. The bottom margin corresponds to the apical boundary of the PP (**B**) or the transition zone SP/CP (**C–I**). Scale bars, 25 µm. **A–I** DL, deep layer; UL, upper layer; PP, preplate; CP, cortical plate; SP, subplate

The first formation of the SVZ in the developing GCR neocortex was noted at GD60 (Fig. 1B). Subsequently, its thickness underwent rapid expansion until GD80, after which it progressively decreases (Fig. 1C-J). Similar to the VZ, Tbr2 + NPCs in the GCR SVZ were detected between GD60-130 (Fig. 1B-H). Using the occurrence of Tbr2 + NPCs as a proxy for estimating the onset and end of cortical neurogenesis [30, 48, 59], the main period of neurogenesis in the GCR neocortex takes place between GD60-130. The number of Tbr2 + NPCs in the SVZ reaches a maximum at GD70-80 (Fig. 1C, D, L), which coincides with that of the SVZ thickness (Fig. 1J). NPCs solely expressing Pax6 (Pax6 + /Tbr2-) were observed in the GCR SVZ at later stages with their number peaking at GD100 (Fig. 2E, L). Between GD80-130, the number of total NPCs in the SVZ exceeds the number of total NPCs detected in the VZ on the same gestational day (Fig. 2K, L).

### Upper and deep layer formation in the prenatal GCR neocortex

Next, we investigated deep and upper layer formation in the developing GCR neocortex and analyzed cortical sections by immunohistochemistry using an antibody for the transcription factor Tbr1, that is characteristically expressed in deep layer neurons (Fig. 2) [30, 49, 84, 122]. This reveals that deep layers, mainly containing Tbr1 + neurons, and upper layers, mainly containing Tbr1– neurons, were established in an “inside-out” fashion with deep layers occurring before upper layers (Fig. 2B-I). The first appearance of Tbr1 + neurons was detected at GD60 (Fig. 2B), which coincides with the first detection of Tbr2 + NPCs in the GCR germinal zones (Fig. 1B, K, L). Tbr1– upper layers first occur in the GCR CP at GD70 and are clearly visible at GD80 (Fig. 2C, D), which coincides with the peak of SVZ thickness and the number of Tbr2 + SVZ NPCs (Fig. 1J, L). After the end of cortical neurogenesis, Tbr1– upper layers occupy a little more than half of the GCR CP (Fig. 2A, H).

### Neuron maturation in the prenatal GCR neocortex

**Fig. 3:**
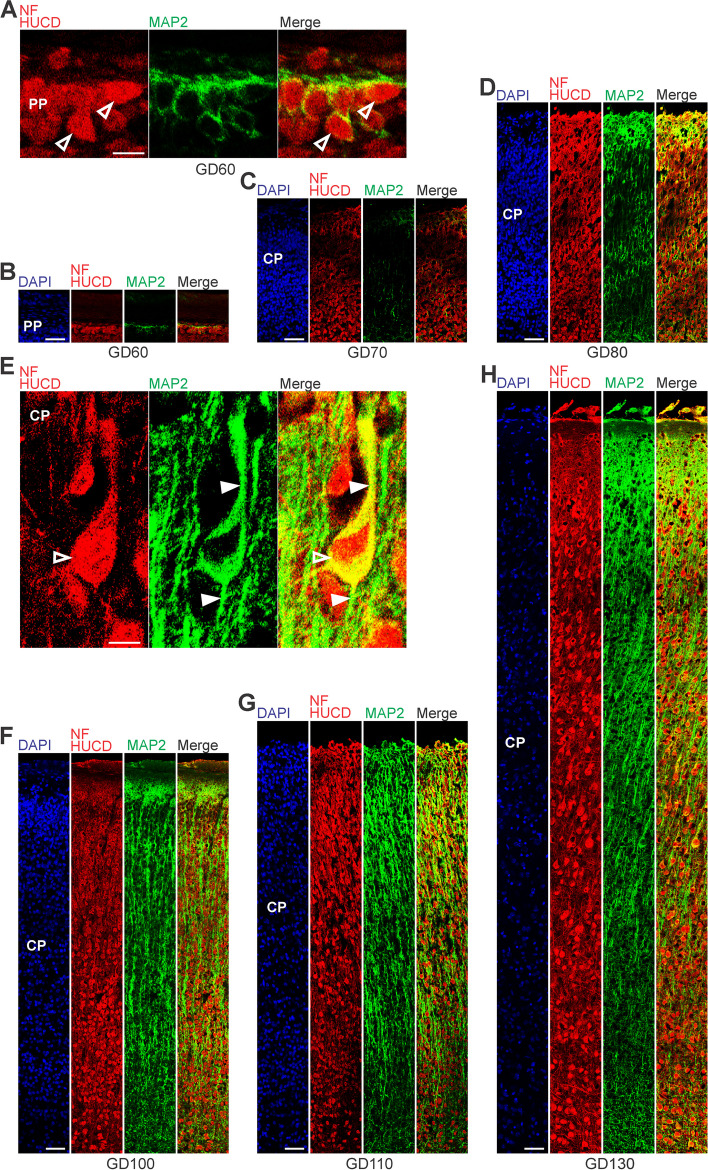
MAP2, Hu C/D and neurofilament expression in the preplate and cortical plate of the developing GCR neocortex. **A–H** Combined immunofluorescence for NF and HUCD (red), and MAP2 (green) and DAPI staining (blue) on 30 µm cryosections of GD60-140 GCR neocortex. **A** Images show neurons with Hu C/D + soma (open arrowhead) in higher magnification of GD60 GCR neocortex. Scale bar, 100 µm. **B–D** The bottom margin corresponds to the apical boundary of the preplate (**B**) or the transition zone SP/CP (**C, D**). Scale bars, 50 µm. **E** Images show neurons with Hu C/D + soma (open arrowhead) and extending Map2 + dendrites (solid arrowhead) in higher magnification of GD130 GCR neocortex. Scale bar, 100 µm. **F–H** The bottom margin corresponds to the transition zone SP/CP. Scale bars, 100 µm. **A–H** PP, preplate; CP, cortical plate; SP, subplate

**Fig. 4:**
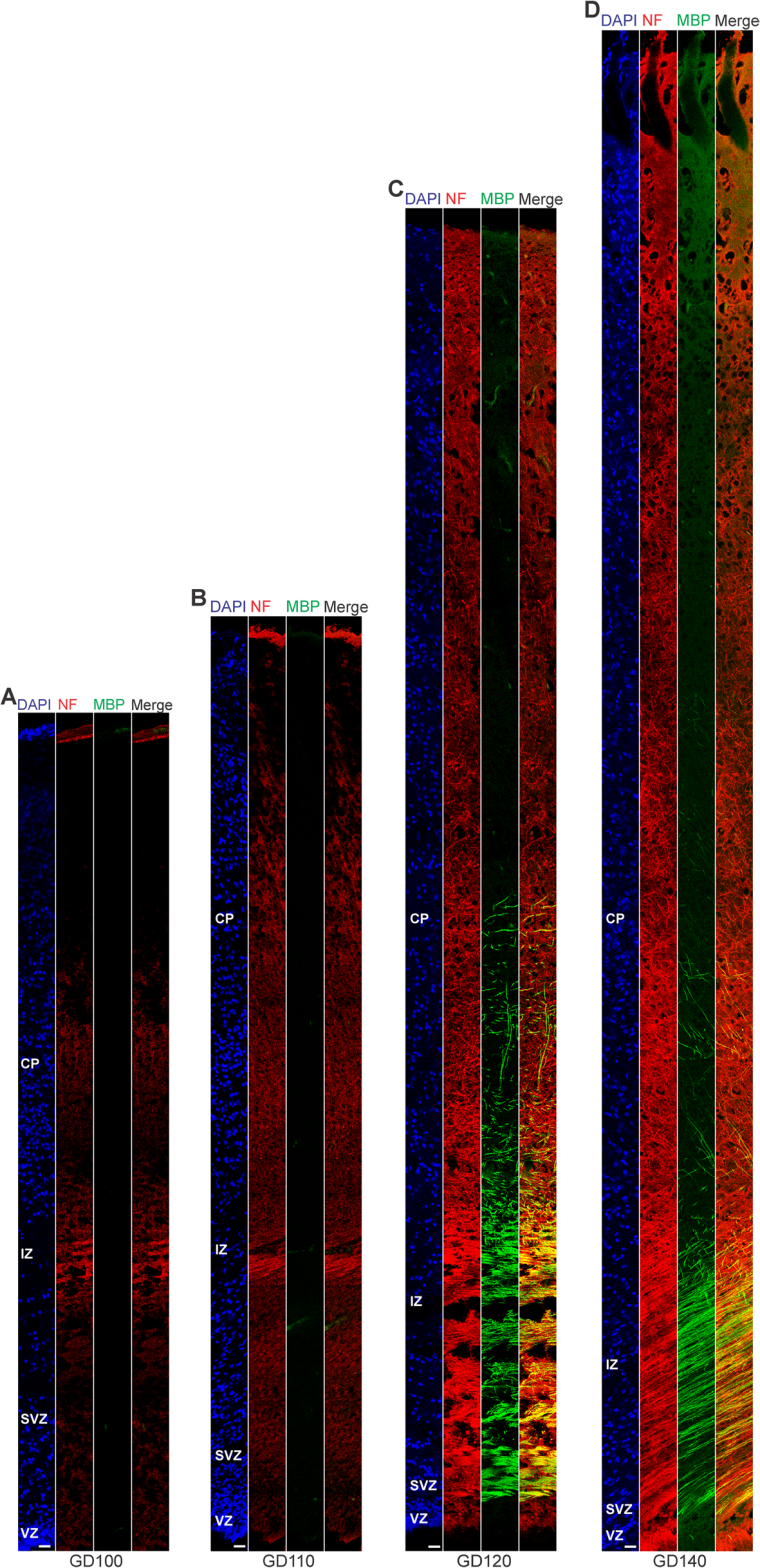
Neurofilament and MBP expression in the cortical wall of the developing GCR neocortex. **A–D**. Double-Immunofluorescence for neurofilament (NF) (red) and MBP (green) and DAPI staining (blue) on 30 µm cryosections of GD100-140 GCR neocortex. The entire cortical wall is shown. VZ, ventricular zone; SVZ, subventricular zone; IZ, intermediate zone; CP, cortical plate. Scale bars, 25 µm

In a next step, we investigated the onset and progression of dendrite and axon formation in the GCR neocortex and analyzed cortical sections by immunohistochemistry using an antibody for the dendrite marker Map2 [11, 20]), the neuronal somatic marker Hu C/D [8, 92] and the axonal marker neurofilament (NF)-H [17, 66, 109] (Fig. 3). The process of axon myelination was examined by double-immunohistochemistry using an antibody for NF-H and myelin basic protein (MBP), which marks myelin sheaths in the central nervous system (Fig. 4) [34, 73, 133]. At GD60, the soma of neurons was entirely stained by Hu C/D and outlined by Map2 immunofluorescence in the GCR neocortex (Fig. 3A, B). Sprouting of Map2 + dendrites first appeared at GD70 (Fig. 3C) and became numerous until GD100 (Fig. 3F). A widespread appearance of NF + axonal structures was first detected between the SVZ and CP, i.e. in the IZ at mid-neurogenesis, i.e. at GD110 (Fig. 4A). These extensions are mostly oriented parallel to the ventricular surface. In the CP, axonal processes, of which many were randomly organized, only become numerous at GD120, and thus at the end of cortical neurogenesis in the GCR. Similarly, widespread myelination was not detected until GD120 in the GCR neocortex, occurring first in the IZ and deep layers of the CP. Prior to birth, i.e. at GD130, neurons in the entire CP showed ramified dendrites and well-delineated axonal processes (Fig. 3H).

### Astrogenesis and oligodendrogenesis in the prenatal GCR neocortex

**Fig. 5:**
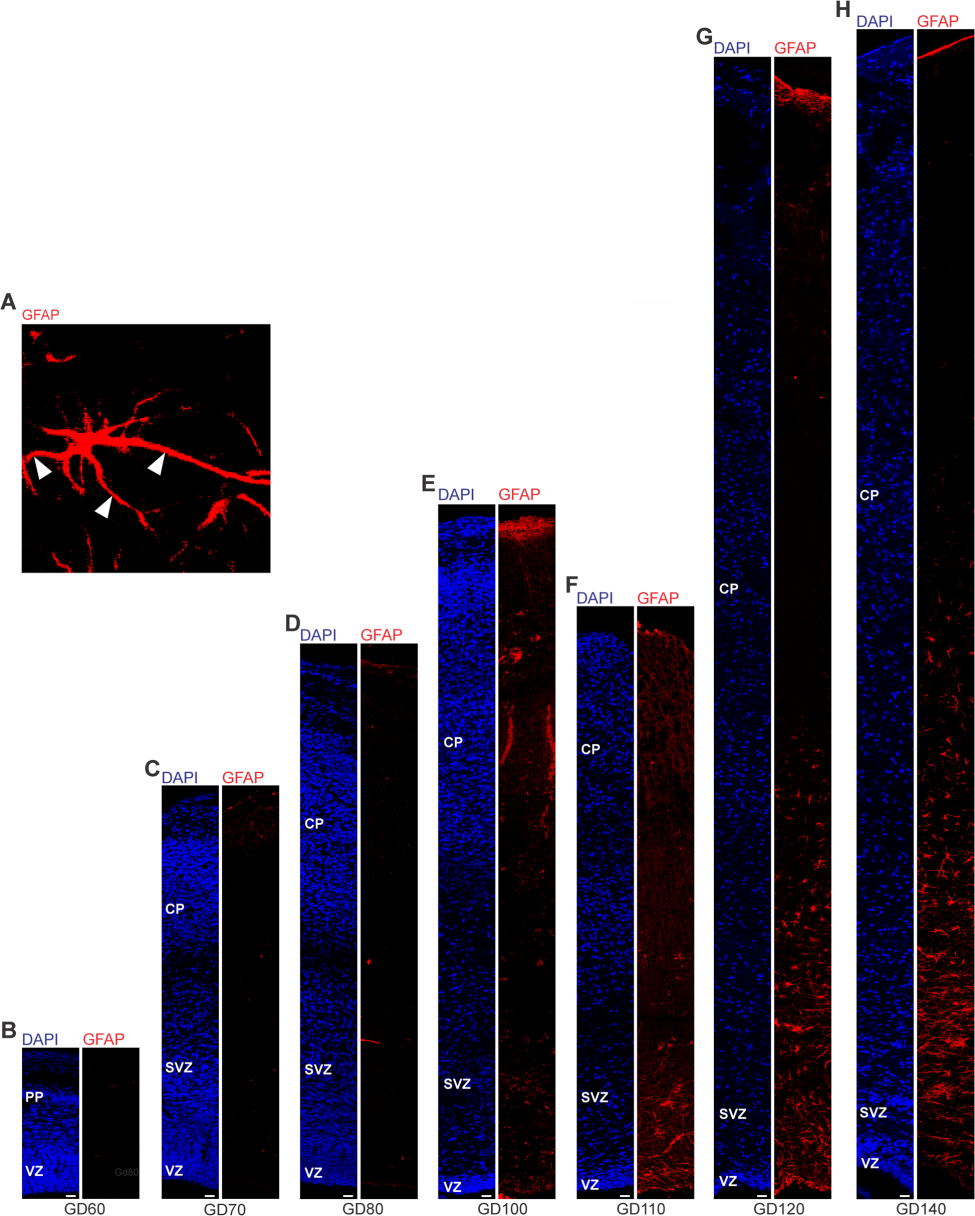
GFAP expression in the cortical wall of the developing GCR neocortex. **A–H**. Immunofluorescence for GFAP (red) and DAPI staining (blue) on 30 µm cryosections of GD60-140 GCR neocortex. (A) Image show GFAP + astrocyte with soma and extending processes (solid arrowhead) in higher magnification of GD120 GCR neocortex. Scale bar, 100 µm. **B–H** The entire cortical wall is shown. Scale bars, 25 µm. VZ, ventricular zone; SVZ, subventricular zone; IZ, intermediate zone; CP, cortical plate

**Fig. 6:**
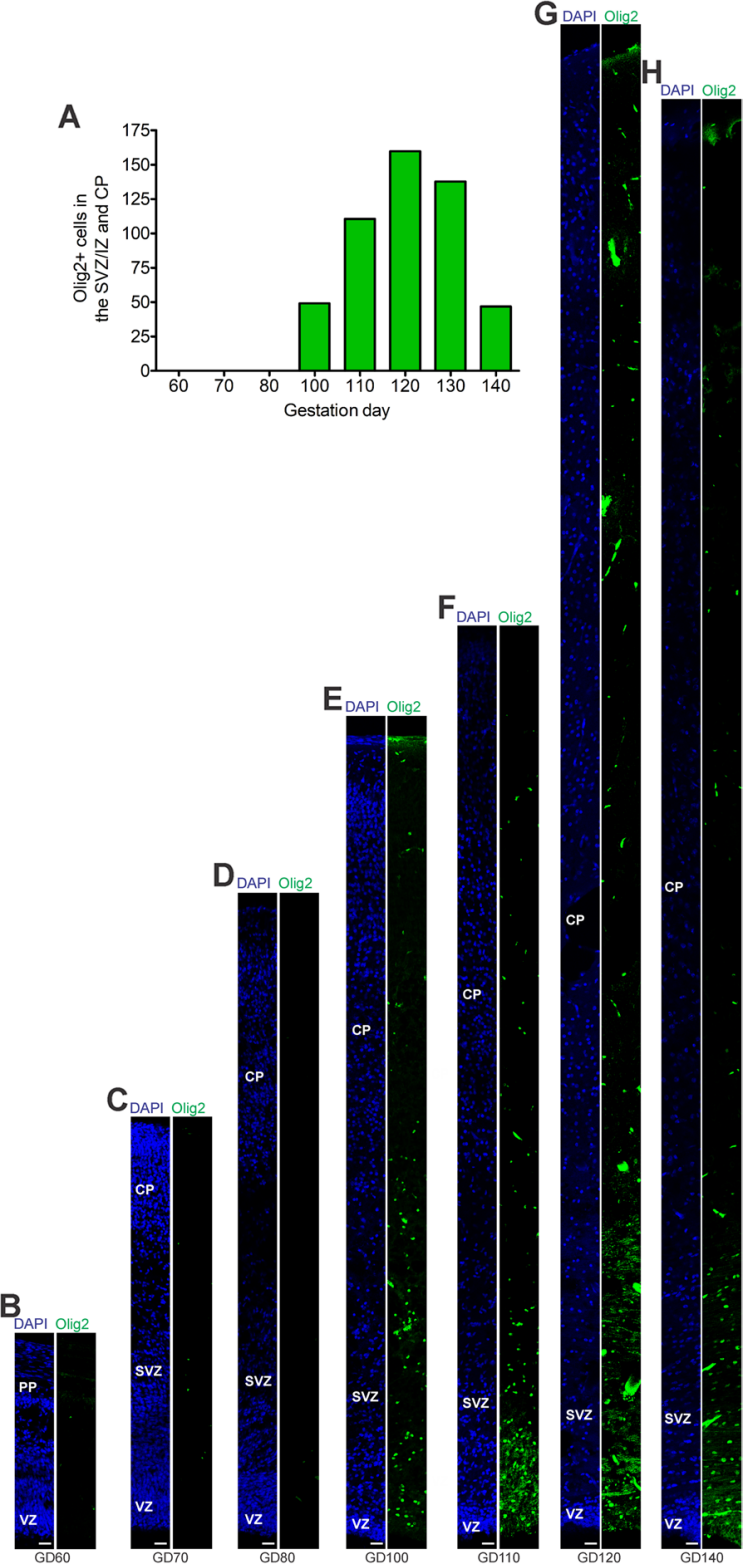
Olig2 expression in the cortical wall of the developing GCR neocortex. **A** Quantification of Olig2 + cells in cortical wall of the GD60-140 GCR neocortex, expressed as number of cells per 100 µm ventricular surface. Cortical wall corresponding to a total ventricular surface of 640–2035 µm was analyzed. Data are from one fetus each. **B–H** Immunofluorescence for Olig2 (green) and DAPI staining (blue) on 30 µm cryosections of GD60-140 GCR neocortex. The entire cortical wall is shown. VZ, ventricular zone; SVZ, subventricular zone; IZ, intermediate zone; CP, cortical plate. Scale bars, 25 µm

We now focused our analysis on the formation of glial cells in the GCR neocortex. In order to visualize astrocytes, we used immunohistochemistry for GFAP, one of their hallmark intermediate filament proteins (Fig. 5) [14, 29, 58]. The first GFAP + structures appeared in the GCR germinal zones during mid-neurogenesis, i.e. at GD100 (Fig. 5E). Mature GFAP + astrocytes, which exhibit a typical star-shaped appearance (Fig. 5A), were first detected in high abundance at GD120 in the GCR neocortex, mainly occurring in the germinal zones, IZ and deep layers of the CP (Fig. 5G). We further investigated the formation of oligodendrocytes in the GCR neocortex and analyzed cortical section by immunohistochemistry for Olig2, a marker for mature oligodendrocytes and their precursors (Fig. 6) [51, 70, 107, 119]. Olig2 + cells first appeared at mid-neurogenesis, i.e. at GD100, in the GCR neocortex with the majority of them populating the germinal zones and IZ, thus likely resembling oligodendrocyte precursors (Fig. 6E). Indeed, oligodendrocytes do not become mature until GD120, a time point when widespread myelination of axons was first detected in the GCR neocortex. The highest number of Olig2 + cells was observed at final stages of neurogenesis in the GCR neocortex (Fig. 6A), when myelination of axons in the GCR CP initiates (Fig. 4C). Together, this indicates that major aspects of astrogenesis and oligodendrogenesis seem to start at the end of neurogenesis in the GCR neocortex.

### Onset of gyrification in the developing GCR

We further examined the onset and the progression of the gyrification of the GCR neocortex (Supplementary Fig. 1). Between GD60-80, the cortical surface appeared smooth and devoid of folds, i.e. gyri and sulci (Supplementary Fig. 1 A-C). Folding of the surface of the GCR neocortex did not start earlier than GD100 (Supplementary Fig. 1D), was clearly visible by GD120, and progresses thereafter (Supplementary Fig. 1F-H). This shows that the process of gyrification is initiated at mid-end neurogenesis in the GCR neocortex, which coincides with the peak of Pax6 + /Tbr2- NPCs in the SVZ (Fig. 1L).

### Comparison of neurogenesis, neuron maturation and gliogenesis to the guinea pig and dwarf rabbit

**Fig. 7:**
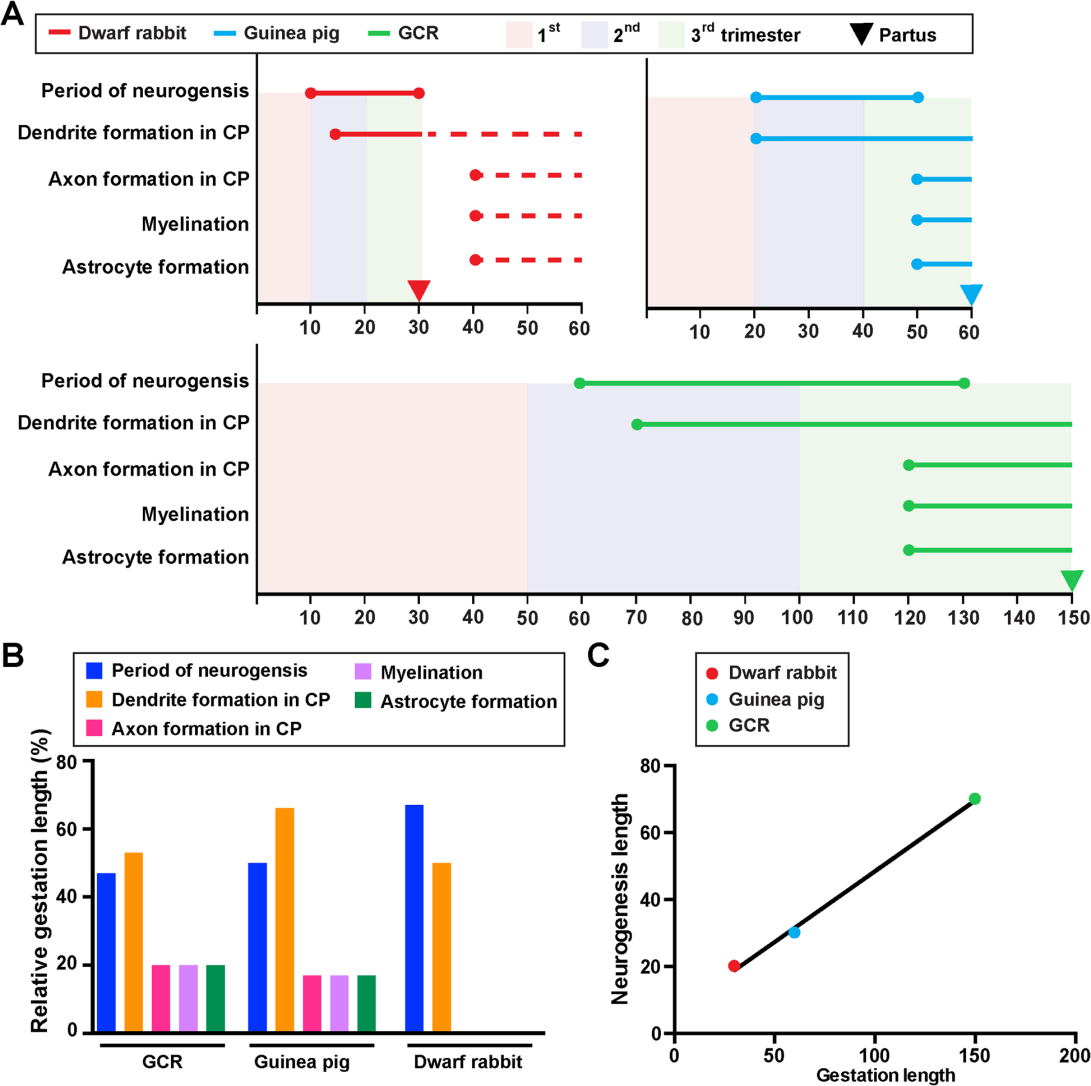
Comparison of specific neurodevelopmental timelines of the neocortex of the GCR, dwarf rabbit and guinea pig neocortex. **A** Onset and duration of the period of neurogenesis, dendrite formation, axon formation, myelination, and astrocyte formation in the developing GCR (green), dwarf rabbit (red) and guinea pig (blue) neocortex. Data for the period of neurogenesis in the GCR are based on the development of Tbr2 + NPCs in the SVZ (Fig. 1); data for dendrite formation, axon formation, myelination, and astrocyte formation in the GCR are based on immunofluorescence staining (Figs. 2, 3, 4 and 5). Data for dwraf rabbit and guinea pig are obtained from the literature [59]. Dashed lines indicate development after birth. Arrows indicate the respective time of birth (partus). CP, cortical plate. **B** Relative lengths of duration of the period of neurogenesis, dendrite formation, axon formation, myelination, and astrocyte formation, expressed as percentage of gestation, in the GCR, dwarf rabbit and guinea pig. **C** Plot of the relationship between the neurogenic period and gestation length, both expressed in absolute days, in the GCR (green), dwarf rabbit (red) and guinea pig (green). The linear regression curve is plotted: y = 0,4231*x + 6,154, *P* < 0.05. Pearson correlation coefficient *r* = 0.9986, *P* = 0.0334

In a next attempt we compared the onset and duration of neurogenesis, neuron maturation and astrogenesis in the GCR neocortex obtained in this study with those of the precocial guinea pig and altricial dwarf rabbit obtained in the same laboratory under similar conditions [59]. Cortical neurogenesis in the GCR does not begin until gestation day 60, while in the guinea pig and dwarf rabbit it begins at a much earlier time point, i.e. on gestation day 10 and 20, respectively (Fig. 7A). When expressed as percentage of gestation, the onset of neurogenesis takes place at a similar time point, i.e. end of first or beginning of second trimester of gestation (Fig. 7A). The main period of cortical neurogenesis covers 70 days in the GCR, which is considerably longer than in the guinea pig and dwarf rabbit, in which it spans only 20 or 30 days, respectively (Fig. 7A. In the GCR, cortical neurogenesis ends before birth, lasting until the middle of the third trimester, which is similar to the guinea pig,however, contrary to the dwarf rabbit, in which it ends just before birth (Fig. 7A). When expressed as percentage of gestation, the main period of cortical neurogenesis takes up about 45% of the gestation length in the GCR neocortex, which is similar to the guinea pig, but contrary to the dwarf rabbit, in which it covers up to 65% of the gestation length (Fig. 7B). When the relationship between the neurogenesis and gestation lengths of all three species was analyzed, we found both parameters to be tightly correlated (*r* = 0.9986, *P* = 0.0334) (Fig. 7C). In all three species, dendrite formation and sprouting begin before birth during early stages of neurogenesis (Fig. 7A). In the GCR and guinea pig CP, the majority of axon formation, myelination and astrocyte formation begins before birth during end stages of neurogenesis, while in the dwarf rabbit CP these processes do not initiate before birth after neurogenesis has terminated (Fig. 7A). In both precocial species analyzed, axon and astrocyte formation, and myelination in the CP take up about 20% of their individual gestation length (Fig. 7B).

## Discussion

This study provides first and detailed data on distinct patterns of prenatal neocortex development, specifically of neurogenesis, gliogenesis and neuron maturation in the neocortex of the GCR, an indigenous African rodent. Moreover, it provides a comparison of the neurodevelopmental milestones between the GCR and closely related precocial and altricial rodents, i.e. the precocial guinea pig and altricial dwarf rabbit.

We show that the GCR exhibits a relatively long period of cortical neurogenesis of 70 days, which is considerably longer when compared to the guinea pig (30 days) and dwarf rabbit (20 days). Similarly, the GCR is characterized by a long gestation length of 150 days, being much longer than in the guinea pig (60 days) and dwarf rabbit (30 days). Previous studies discussed a close relationship between the lengths of neurogenesis and gestation [38, 59, 69, 113]. Indeed, we found these two processes to be tightly correlated in the three species analyzed. Furthermore, the neurogenic period in the GCR was quite similar to that of macaque (~ 67 days), which also exhibits a similar gestation length (~ 165 d) as the GCR. Compared to humans, the neurogenesis and gestation length in the GCR are about half as long in the GCR [12, 31, 44, 101, 112, 120, 131]. Moreover, we show that cortical neurogenesis in the GCR starts at GD60, and thus approximately 40 or 50 days later when compared with the dwarf rabbit and guinea pig, respectively. However, when expressed in percentage of gestation, the onset of neurogenesis occurs at a similar developmental time point, i.e. at the very end of the first or beginning of the second trimester of gestation in the three species analyzed. Interestingly, cortical neurogenesis in the sheep, which exhibits a similar gestation length (~ 150 days) as the GCR, has been reported to start at GD60, i.e. in the beginning of the second trimester [38]. Moreover, in primates, specifically marmoset, macaque and human, cortical neurogenesis starts in the early second trimester of gestation [12, 31, 44, 60, 120, 131]. Together this indicates that the length as well as the onset of neurogenesis might be linked to gestation length.

The length of the neurogenic period has been noted as one of the key determinants defining neuronal output and thus brain size [33, 113]. Thus, this higher cortical neurogenesis length could contribute to the more expanded brain of the GCR, possessing an average adult brain mass of 12.22 g [54], than the guinea pig and dwarf rabbit with an average adult brain mass of 4.3 and 9.6 g, respectively [59]. However, the longer period of neurogenesis in the GCR does not appear to result in a higher relative brain size, as its encephalization quotient, defined as the ratio between observed to predicted brain mass for an animal of a given size, is 0.49 [54], which is similar to that of the guinea pig (0.46), but lower than that of the dwarf rabbit (0.72) [59]. Together, this provides evidence for the notion that total neurogenesis length is one important factor contributing to (absolute) brain size expansion. Other factors may include the absolute number of NPCs, the relative abundance of each NPC type and their cell cycle length [91, 98, 114, 117, 118].

Our data reveal, that the number of total NPCs in the SVZ is higher than in the VZ of the same gestational day between GD80-130, thus endowing the SVZ with the major NPC pool during mid-end stages of neurogenesis in the GCR neocortex. These data are in line with findings in primates including marmoset, macaque and human and other species exhibiting a highly expanded neocortex such as ferret, but are in contrast to that of other rodents including mouse, rat and guinea pig, in which the VZ constitutes the major NPC pool throughout development [10, 12, 31, 44, 60, 63, 64, 78, 103, 112]. Moreover, at mid-end stages of neurogenesis, i.e. between gestation days 100–130, Pax6 + (Tbr2 + /Tbr2-) NCPs constitute the major BP subtype in the GCR SVZ. Given that sustainment of Pax6 expression in NPCs is linked to higher cell proliferation, our data suggest that similar to primates such as marmoset, macaque and human and ferret, the developing GCR neocortex contains highly proliferative BPs including bRG in high abundance at mid-end stages of neurogenesis [12, 44, 60, 130]. Previous studies have linked the basal process scaffold of bRG to the tangential dispersion of radially migrating neurons, resulting in the tangential expansion of the neocortex and thus the formation of gyri and sulci [103]. Indeed, our data show that the abundant occurrence of Pax6 + BPs in the SVZ coincides with the onset of surface folding of the GCR neocortex. Future studies will address the morphology, mode and rate of cell division of distinct NPC subtypes to precisely evaluate their contribution to neocortex development in the GCR.

Similar to other mammalian species, deep- and upper projection neurons are sequentially produced in an '*inside-out*' fashion in the GCR neocortex [115, 122, 123]. At the end of cortical neurogenesis, Tbr1- upper layers occupy a little more than half of the GCR CP (Fig. 2A, H), which is also consistent with observations in primates including human, cat, pig and cattle and tammar wallaby [23, 38, 105]. After the completion of neuronal migration, dendritic and axonal sprouting, which are meticulously-controlled programmed events, occur in order to ensure proper neural circuit assembly and connectivity during development [36, 50]. The first widespread appearance of NF + axonal structures was first detected in the IZ at mid-end stages neurogenesis, i.e. at GD110 (Fig. 4A). As these extensions are mostly oriented parallel to the ventricular surface, they might represent interneurons, which migrate tangentially from the ventral into the dorsal telencephalon during prenatal development [21, 22, 43, 67, 68, 75, 129]. In the GCR CP, we show that—similar to other mammals—dendrite formation precedes axon formation [9, 110, 134]. Consistent with findings in primates including macaque and human, major aspects of neuron maturation and gliogenesis in the GCR CP starts long before birth [102, 132]. As a result, the GCR neocortex exhibits a relatively high maturation status, seemingly containing neurons with well-developed dendrites and myelinated axons and astrocytes, at the time of birth. This indicates that the GCR offspring has acquired the morphological prerequisites to reach its high functional state, characteristic of precocial animals, at birth. As such, our data confirm the neurodevelopmental gradient along the altricial-precocial spectrum, with a greater proportion of neocortex growth and maturation taking place during prenatal development in precocial mammals as opposed to altricial mammals, in which these processes largely occur during early postnatal life. In order to demonstrate whether the existing neurons in the prenatal GCR are indeed genuine neurons, and thus functionally mature, additional set of immunohistochemical markers of neuronal maturation as well as electrophysiological techniques, i.e. patch clamp, will be used in future studies.

Because of the short life span (4 years), high reproductivity with an average litter size of 2 to 6, easy accessibility as an indigenous African rodent [3, 86, 87, 93], the GCR is fast gaining the attention of African researchers in a bid to develop natural and locally sourced laboratory models for neuroscience-based research, including neurodevelopmental and eco-toxicological studies [6, 86, 87]. Importantly, we show that the GCR shares some similarities with primate neocortex development, i.e. an expanded SVZ and an abundant occurrence of Pax6 + BPs during mid-neurogenesis, which takes place during mid-gestation, and a relatively high degree of maturation at the time of birth. Therefore, our study strongly warrants further analysis in order to find out whether the GCR appears to be more suitable than other rodents including mouse and rat to model and understand specific aspects of primate neocortex developmental and developmental brain disorders. In this regard, the specific data of the distinct neurodevelopmental events in the prenatal GCR, i.e. the onset and duration of neurogenesis, neuron maturation and gliogenesis, provided by this study may serve as empirical reference data and foundation in future studies. Together, this work deepens our understanding of neocortex development of the GCR, of the biodiversity and phylogeny of this hystricomorph rodent and of the evolution of mammalian altriciality and precociality.

### Supplementary Information

**Additional file 1:** **Supplementary Fig. 1.** Development of gyrification of the GCR neocortex. (A-H) Dorsal view of the gross morphological features of the developing GCR brains. Arrowheads indicate folding of the cortical surface. Scale bars, 2.5 mm.

## Data Availability

The datasets used and/or analysed during the current study are available from the corresponding author on reasonable request.

## Abbreviations

AP: Apical progenitor
bRG: Basal radial glia
BP: Basal progenitor
CP: Cortical plate
GCR: Greater cane rat
GD: Gestation day
NF: Neurofilament
IZ: Intermediate zone
NPC: Neural stem and progenitor cells
MBP: Myelin basic protein
PBS: Phosphate-buffered saline
PP: Preplate
SP: Subplate
SVZ: Subventricular zone
VZ: Ventricular zone
