# Supplementary figures and images for: Neocortex neurogenesis and maturation in the African greater cane rat
Source: Neural Dev. 2023 Oct 13;18:7. doi: 10.1186/s13064-023-00175-x (PMC10571270; doi:10.1186/s13064-023-00175-x)

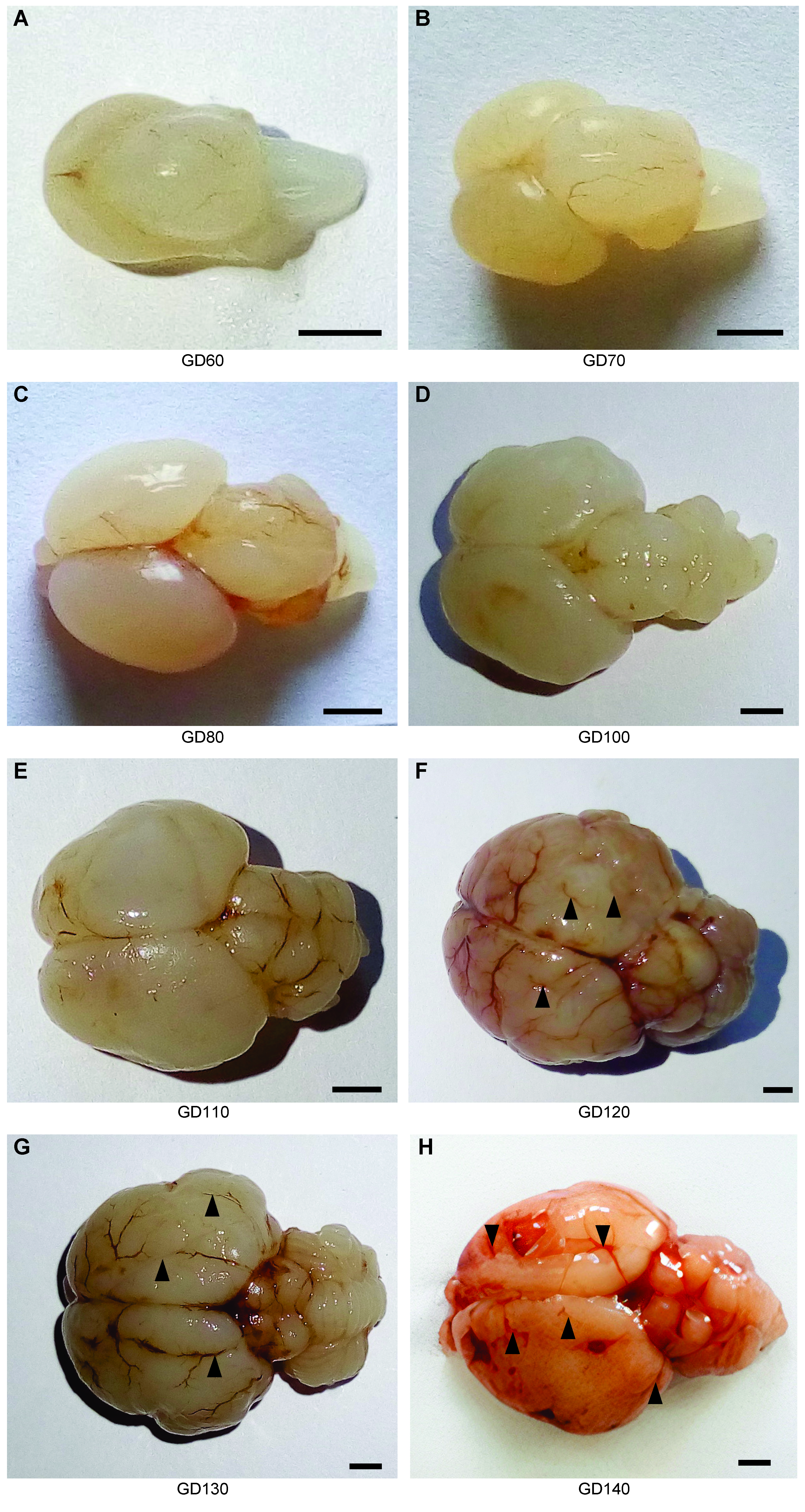

Supplement: Supplementary file 1 — Additional file 1: Supplementary Fig. 1. Development of gyrification of the GCR neocortex. (A-H) Dorsal view of the gross morphological features of the developing GCR brains. Arrowheads indicate folding of the cortical surface. Scale bars, 2.5 mm. [file 13064_2023_175_MOESM1_ESM.tif]
